# Splenic Vein Embolization Using Coil Anchors and Prophylactic Occlusion of a Hepatofugal Collateral for Hepatic Encephalopathy due to Splenorenal Shunt: Technical Note and Literature Review

**DOI:** 10.1155/2013/160653

**Published:** 2013-03-27

**Authors:** Masayoshi Inoue, Toshihiro Tanaka, Hiroyuki Nakagawa, Tetsuya Yoshioka, Kimihiko Kichikawa

**Affiliations:** ^1^Department of Radiology, Nara Prefectural Nara Hospital, 1-38-1 Hiramatu, Nara 631-0846, Japan; ^2^Department of Radiology, Nara Medical University, 840 Shijo-cho, Kashihara 634-8522, Japan; ^3^Department of Radiology, Narumi Hospital, 19 Shinagawa-cho, Hirosaki 036-8183, Japan

## Abstract

*Purpose*. Interventional treatment strategies for patients with encephalopathy due to splenorenal shunt remain controversial. Portosplenic blood flow separation by occluding the splenic vein could avoid the complication of severe portal hypertension, but it would require repeated reintervention due to recurrence of symptoms. This paper describes occlusion of the splenic vein using coil anchors and prophylactic embolization of a collateral hepatofugal vessel with no recurrence of hyperammonemia. *Materials and Methods*. A 51-year-old woman with severe cirrhosis had hepatic encephalopathy due to a large splenorenal shunt. The serum ammonia level was 132 **μ**g/dL. Via a transileocolic approach, the splenic vein was completely embolized with 0.035-inch metallic coils using coil anchors while preserving the splenorenal shunt. In addition, one of the collateral vessels of the portal vein, the retrogastric vein, was also embolized prophylactically. *Results*. After this procedure, the serum ammonia level decreased immediately to 24 **μ**g/dL. The portal venous pressure increased by only 1.5 mmHg. Hepatic encephalopathy had not been observed for 25 months after the procedure, and neither retention of ascites nor worsening of esophageal varices and liver function was observed. *Conclusion*. This procedure appears to be safe and effective for hepatic encephalopathy caused by a splenorenal shunt.

## 1. Introduction

Hepatic encephalopathy due to hyperammonemia is a critical problem in patients with liver cirrhosis. Portosystemic communication through a splenorenal shunt is a common cause of hyperammonemia. For these patients, surgical shunt ligation has been conducted, but with a high mortality rate [1]. Since 1984, interventional management strategies for splenorenal shunt have been reported [2]. In these articles, shunt obliteration using ethanolamine oleate and shunt embolization using coils have been the main techniques [2–11]. However, theoretically, direct shunt occlusion could cause severe portal hypertension. In 2004, Zamora et al. first reported portosplenic blood flow separation by occluding the splenic vein [12]. They suggested that this technique was safe, avoiding severe portal hypertension, but it required repeated reintervention because hyperammonemia frequently recurred due to recanalization of the splenic vein and development of collateral hepatofugal flows.

 This paper describes our technique of portosplenic blood flow separation, presenting a case with hepatic encephalopathy due to a splenorenal shunt successfully treated by complete occlusion of the splenic vein using coil anchors and embolization of a collateral hepatofugal vessel to prevent recurrence of hyperammonemia.

 In addition, the published literature regarding interventional management of splenorenal shunt in the Medline database is reviewed, and the advantages and disadvantages of each technique are discussed.

## 2. Case Report

A 51-year-old woman with viral C-type liver cirrhosis, in whom hepatocellular carcinoma (HCC) had been treated with radiofrequency tumor ablation and percutaneous ethanol injection one year earlier, was admitted to our institute with a disturbance of consciousness. Laboratory tests on admission revealed a serum ammonia level of 132 *μ*g/dL, total bilirubin of 2.77 mg/dL, prothrombin time of 72.7%, and albumin of 2.8 mg/dL. The patient was diagnosed as having hepatic encephalopathy and severe liver dysfunction. Hepatic encephalopathy was assessed and evaluated according to the Sherlock's classification [13], and the severity of liver disease according to the Child-Pugh score was 9 (class B) [14]. An abdominal computed tomography scan demonstrated a massive splenorenal shunt and shrinkage of the portal vein with a small amount of thrombus. Conservative therapy with dietetic therapy and amino acid preparations was performed, but unsuccessfully.

**Figure 1:**
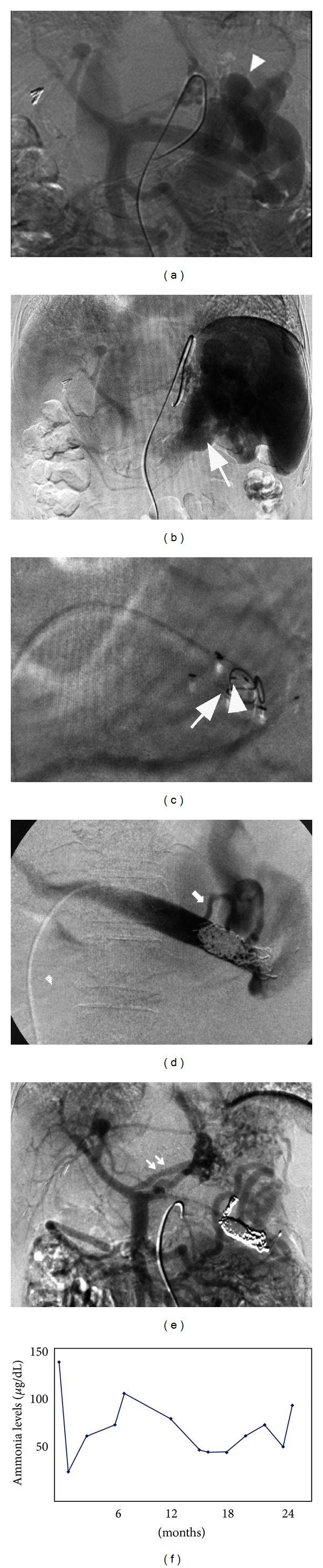
(a) Portography from the superior mesenteric artery shows the hepatofugal flow in the splenic vein and a large splenorenal shunt (arrowhead). (b) The venous phase of a selective splenic arteriogram shows that the splenic venous blood flows into the left renal vein via the splenorenal shunt (arrow). (c) A device (Coil Anchor, Medikit Co., Miyazaki, Japan) (arrowhead) to prevent migration of the coils has been placed. After the placement of this device, one 0.035-inch platinum coil was placed (arrow). (d) Selective splenovenography after embolization of the splenic vein shows the development of a hepatofugal collateral, retrogastric vein (arrow), connected to the splenorenal shunt. This collateral was embolized with 2 microcoils. (e) Portography from the superior mesenteric artery after embolization demonstrates a clear depiction of portal branches and no splenorenal shunt. There was hepatofugal flow via the coronary vein (double arrow), but it was not embolized to avoid increasing portal pressure. (f) Venous blood ammonia levels measured after the procedure. The patient had encountered no symptoms of hepatic coma for 24 months after procedure.

The flow directions of the superior mesenteric vein and the splenic vein were evaluated by angiography. Portography via superior mesenteric arteriography demonstrated that most of the superior mesenteric venous flow was hepatofugal and entered the splenorenal shunt (Figure 1(a)). Selective splenic arteriography showed that all splenic venous flow entered the splenorenal shunt (Figure 1(b)).The diameters of the splenic vein and the splenorenal shunt were 14 mm and 18 mm, respectively. Because the patient's liver function was poor, there was a concern about the increase in portal vein pressure, which could cause liver failure if obliteration or occlusion of the splenorenal shunt was to be performed. Therefore, portosplenic blood flow separation by occluding the splenic vein was performed.

The procedure was performed transileocecally because the intrahepatic portal vein branches were very narrow, and percutaneous transhepatic puncture could have been difficult. Laparotomy was performed under general anesthesia. A 5.5 Fr introducer sheath was inserted into the ileocecal vein. After obtaining the direct superior mesenteric venography, splenic vein embolization was performed. First, a 5.5 Fr hook-shaped angiographic catheter (Cook, Bloomington, IL, USA) was advanced into the splenic vein close to the entry point of the splenorenal shunt. Prior to coil embolization, coil anchors (Coil Anchor II; Medikit Co., Tokyo, Japan) were placed. The characteristics of the coil anchor were as follows: (i) w-shaped device made of stainless steel; (ii) it can be deployed through an angiographic catheter with an inner diameter of 0.038 inch; and (iii) the type L coil anchor has a width of 23 mm and a length of 23 mm, and it can be adapted up to a vessel diameter of 18 mm. To form the framework to prevent coil migration, a total of 4 coil anchors were deployed, which allowed the placement of smaller coils into the large vascular structure to occlude it. A total of 21 0.035-inch platinum coils (Tornado; Cook, Bloomington, IL, USA), with a tapering diameter of 10 mm–5 mm were placed. The coils were successfully trapped by the coil anchors and did not migrate (Figure 1(c)). After complete embolization of the splenic vein, superior mesenteric venography showed several hepatofugal collateral flows, including the left, right, and retrogastric veins. We hypothesized that if all of the collateral vessels were to be occluded, the portal vein pressure would increase. Therefore, only the retrogastric vein was embolized (Figure 1(d)). Consequently, the portal vein pressure increased by only 1.5 mmHg (from 10 mmHg to 11.5 mmHg). The final superior mesenteric venography showed that the left and right gastric veins maintained hepatofugal flows (Figure 1(e)). The blood ammonia level decreased to 24 *μ*g/dL immediately after the procedure. The patient survived for 25 months without recurrence of encephalopathy and required no reinterventions (Figure 1(f)), and neither retention of ascites nor worsening of esophageal varices was observed. She died due to HCC rupture, which was unrelated to the portosystemic shunt.

## 3. Literature Review

All English-language articles describing interventional treatment for patients with hepatic encephalopathy due to splenorenal shunt were searched in the Medline database, and all relevant references cited in the identified articles were reviewed [2–12, 15]. Twelve articles, published from 1984 to 2007, were found (Table 1). All reports were either case reports or technical notes, in which a total of 26 cases were reported; there were no systematic studies or review articles. These techniques could be classified into three groups: (i) balloon-occluded retrograde transvenous obliteration (BRTO), 11 cases in 6 articles; (ii) shunt embolization, 7 cases in 5 articles; and (iii) portosplenic blood flow separation, 7 cases in 2 articles. BRTO and shunt embolization occlude the splenorenal shunt, which causes the splenic venous flow to enter into the portal vein. In contrast, portosplenic blood flow separation occludes the splenic vein, which preserves the shunt and prevents the splenic venous flow from entering the portal vein. The portal venous pressures measured after shunt embolization showed an increased pressure of 8 mmHg, whereas those after splenic vein embolization showed an elevation of pressure up to 4 mmHg. In 3 cases treated by BRTO and in 6 cases treated by shunt embolization, there were reported complications of liver dysfunction and progression of esophageal varices, ascites, and pleural effusion, including 2 fatal cases, whereas no complications were reported in 7 cases treated by splenic vein embolization. Recurrence of hepatic encephalopathy was reported in one case, 12 months after BRTO; in 2 cases, 3 and 6 months, respectively, after shunt embolization; and in 3 cases, 12 days, 14 months, and 16 months, respectively, after splenic vein embolization.

## 4. Discussion

For a patient with encephalopathy caused by a large splenorenal shunt, surgical treatment such as shunt ligation or a combination of shunt closure and splenectomy has been performed conventionally. However, due to the unacceptably high mortality rate (40%) caused by surgical procedures [1], interventional radiology has recently been performed as a less invasive therapy with the expectation that it would be safe and effective. Since the introduction of interventional shunt occlusion using a detachable balloon in 1984, all previous published articles have been case reports. To date, there is no agreed standard technique, and the treatment strategies remain controversial.

The important advantage of splenic vein embolization with preservation of the portosystemic shunt is to avoid complication risks caused by extreme elevations of portal venous pressure. This treatment separates the hepatopetal splenic venous flow from the portal venous flow via the superior mesenteric vein. The splenic vein and the collateral vessels from the portal vein flow into the splenorenal shunt. The present literature review demonstrates that splenic vein embolization showed minimal elevation of portal vein pressure (up to 4 mmHg versus over 8 mmHg by shunt occlusion). In addition, no complications were reported with splenic vein embolization, whereas liver dysfunction and progression of esophageal varices and ascites, including two fatal cases, were reported with shunt occlusion. In the present case, due to the patient's poor liver function, there was a high risk of liver failure if we would have occluded the splenorenal shunt. Therefore, we chose splenic vein embolization with the preservation of the splenorenal shunt and successfully treated it with a slight elevation (1.5 mmHg) of portal vein pressure.

 The major disadvantage of the splenic vein embolization previously reported by Zamora et al. was the repeated reintervention for recurrent encephalopathy. They reported that reinterventional procedures were conducted 3 times during the first 5 months after the first splenic vein embolization. This was due to the recurrence of symptoms caused by the incomplete embolization of the splenic vein and the development of collateral hepatofugal flows. To overcome these problems, we adopted the following techniques. First, we used coil anchors to embolize the splenic vein, because the splenic venous flow, which connects to a large shunt, is quite rapid. The risk of coil migration and incomplete occlusion would be high if we were to use microcoils, including detachable coils. Coil anchors allowed the high flow of the splenic vein to be embolized with 0.035-inch fiber coils without any migration. Previously, Mori et al. reported the usefulness of coil anchors in the embolization of pulmonary arteriovenous malformation [16]. We considered that embolization of the high flow splenic vein is also a good indication for this device. Second, we embolized the retrogastric vein at the same time. In patients with liver cirrhosis, the left, right, and retrogastric veins often become the collateral hepatofugal flows of the portal vein. These collateral vessels have an important role to prevent elevation of portal vein pressure, but they could cause the recurrence of hyperammonemia. In this case, we embolized one of the collateral vessels and eventually prevented both the elevation of portal vein pressure and the recurrence of encephalopathy. The reasons why we chose to embolize the retrogastric vein were as follows: (i) the connection route between the retrogastric vein and the splenorenal shunt was shorter than the others, and there was a possibility that the hepatofugal flow of the retrogastric vein could cause the recurrence of hyperammonemia; and (ii) the retrogastric vein was smaller than the others, and the occlusion of the retrogastric vein could be safer, without any elevation of the portal vein pressure. Indeed, it is difficult to predict how many and which collateral vessels should be occluded. If a major collateral vessel would be embolized with coils, and the portal vessel pressure would increase extremely, it could become impossible to remove the coils to recanalize the collateral vessel. A temporary occlusion test using a balloon catheter is a promising option, but two catheters, including a balloon catheter and a pressure measurement catheter, must be inserted into the portal vein. We chose not to adopt this method because the ileocecal vein was too narrow to insert two catheters.

 Regarding access to the splenic vein, it is difficult to puncture the slender portal vein transhepatically due to severe cirrhosis. Therefore, we adopted the transileocecal approach, which was technically easier than portal vein puncture, although general anesthesia was required. This approach has been frequently reported in portal vein embolization before hepatectomy and treatment of gastric varices [17]. Our technique using the transileocecal approach for splenic vein embolization is the first to be reported.

 In conclusion, our technique of embolization using coil anchors achieved complete occlusion of the high flow splenic vein, and prophylactic embolization of the hepatofugal collateral prevented the recurrence of hyperammonemia. Further research including a greater number of cases is necessary to evaluate the effect of this method.

## Figures and Tables

**Table 1:** Previous reports regarding interventional treatment for splenorenal shunt.

Authors	Year	Number	Method (cases)	Embolic material	Recurrence of coma	Complications
Vavasseur et al. [3]	1994	1	SE	MC, NBCA	(−)	Esophageal varix
Shioyama et al. [4]	1996	1	BRTO	EOI	(−)	
Numata et al. [5]	1998	1	BRTO	EOI	(−)	
Sakurabayashi et al. [6]	1997	5	SE or BRTO	MC, EOI	(+)	Ascites
Takashimizu et al. [7]	2007	1	BRTO	EOI	(−)	Ascites
Potts et al. [2]	1984	1	SE	DB	(−)	
Zamora et al. [12]	2004	1	Separation	MC	(+)	
Mezawa et al. [15]	2004	6	Separation	MC	(+)	
Clarke et al. [8]	1989	1	SE	MC	(−)	
Uflecker et al. [9]	1987	2	SE	MC	(−)	Intraabdominal bleeding (died) and esophageal varix rupture
Matake et al. [10]	2007	1	BRTO	EOI	(+)	
Miyamoto et al. [11]	2003	5	BRTO	EOI	(−)	
Ours	2011	1	Separation	MC	(−)	

SE: shunt embolization, BRTO: balloon-occluded retrograde transvenous obliteration, MC: metallic coil, EOI: ethanolamine oleate, and DB: detachable balloon.
